# Incidence of Second Primary Malignancies in Patients with Castration-Resistant Prostate Cancer: An Observational Retrospective Cohort Study in the United States

**DOI:** 10.1155/2019/4387415

**Published:** 2019-02-11

**Authors:** Catherine W. Saltus, Zdravko P. Vassilev, Jihong Zong, Brian Calingaert, Elizabeth B. Andrews, Montse Soriano-Gabarró, James A. Kaye

**Affiliations:** ^1^RTI Health Solutions, Waltham, Massachusetts, USA; ^2^Bayer US, Whippany, New Jersey, USA; ^3^RTI Health Solutions, Research Triangle Park, North Carolina, USA; ^4^Bayer AG, Berlin, Germany

## Abstract

**Background:**

New therapies for castration-resistant prostate cancer (CRPC) may be associated with increased risk of second primary malignancies (SPM). We therefore estimated the population-based incidence of SPM among patients with CRPC in the Surveillance, Epidemiology, and End Results (SEER)-Medicare database. We also estimated the proportion of men with CRPC with bone metastases and overall survival.

**Methods:**

We conducted a retrospective cohort study of United States (US) men aged ≥ 65 years with CRPC. Cohort entry was from January 1, 2000, to December 31, 2011, with follow-up through December 31, 2013. Castration resistance was defined by treatment with second-line systemic therapy (after surgical or medical castration). SPM were diagnoses of primary cancers (other than prostate) in SEER or Medicare data.

**Results:**

Altogether 2,234 patients met eligibility criteria. Most (1,887; 84.5%) had evidence of bone metastases in Medicare claims. SPM occurred in 172 patients (incidence rate 5.9 per 100 person-years; 95% confidence interval [CI], 5.0-6.8; standardized incidence ratio = 3.1, 95% CI, 2.8-3.6, based on SEER incidence rate of all malignancies except prostate cancer among men aged ≥ 65 years). The most common SPM were lung/bronchus (n = 29, 16.9%), urinary bladder (n = 22, 12.8%), and colon/rectum (n = 21, 12.2%). Median survival was 1.2 years (95% CI, 1.1-1.3); 5-year survival was 9% (95% CI, 7-11%).

**Conclusions:**

This study provides the first estimate of SPM risk in older men with CRPC in the US. The incidence rate is approximately threefold higher than the population-based cancer incidence among men without prostate cancer.

## 1. Introduction

New systemic therapies have been introduced for the treatment of castration-resistant prostate cancer (CRPC) in recent years, some of which may be associated with an increased risk of second primary malignancies (SPM). Although SPM can occur in patients with cancer irrespective of treatment, long-term cumulative radiation exposure may be associated with an increased risk of cancer [1], and radiopharmaceutical therapy of bone metastases may contribute to a patient's overall radiation exposure. This could raise concern about the possibility of induction of SPM in men with CRPC.

Population-based epidemiological studies have been conducted to estimate the incidence rates of SPM among patients with cancer, including those with prostate cancer [2, 3]. However, such data for patients with CRPC are limited. We therefore conducted a retrospective cohort study of SPM incidence among men with CRPC in the United States (US) using the National Cancer Institute's (NCI's) Surveillance, Epidemiology, and End Results (SEER)-Medicare database. The SEER-Medicare database population is representative of the general US population and is the largest available source of detailed population-based medical information on men aged 65 years or older with prostate cancer [4].

In clinical practice, CRPC is defined as progression of advanced prostate cancer despite medical or surgical castration. Key defining factors include castrate level of serum testosterone plus evidence of progression, either biochemically (serial prostate-specific antigen assays) or by radiologic evaluation (bone scan or measurable soft tissue masses). However, information regarding serum testosterone levels, prostate-specific antigen measurements, and results of imaging studies are not available in Medicare claims data [5]. Therefore, the present study used a pragmatic approach for defining CRPC based on “second-line” treatments administered after surgical or medical castration to indicate that progression had occurred despite castration.

The primary objective of this study was to estimate the population-based incidence rate of SPM among patients with CRPC. Secondary objectives were to estimate the proportion of men with CRPC who had evidence of bone metastases and to estimate overall survival of men with CRPC.

## 2. Methods

### 2.1. Study Period

The study period was 01 January 2000 through the latest year of available Medicare data (31 Dec 2013).

### 2.2. Data Source

The source of data for this study was the SEER-Medicare linked database, administered by the US NCI, which at the time this study was conducted contained SEER data from 1991 through 2011 and linked Medicare data through 2013. This source combines data from the SEER Program, which collects population-based cancer registry data covering approximately 28% of the US population for the diagnosis years included in this study, with data from Medicare, the US federal health insurance program primarily for people aged 65 years and older [4]. It contains detailed information for each primary cancer and individual, including the initial diagnosis and date of death. “SEER-Medicare data” refers to a series of files: one contains SEER data, while the others contain Medicare claims data in separate files for specific types of services (e.g., hospital, physician, or outpatient visits). Patient data are linked across the various files using the unique SEER case identification number [6].

### 2.3. Study Design and Subjects

This was a retrospective, observational cohort study of men in the US aged 65 years or older with CRPC.

SEER data were used initially to identify all men in the study population diagnosed with prostate cancer (primary site code of prostate cancer [International Classification of Diseases for Oncology, Third Edition topography code C61.9] with behavior code “/3” [malignant] in SEER data) from 2000 to the end of available data (2011). Then, Medicare data were used to identify surgical castration (bilateral orchiectomy) by applying an algorithm specifying orchiectomy [7]. A list of drugs described in the American Urological Association Guidelines [8] was adapted to identify medical castration (androgen deprivation therapy) in Part D data or corresponding Healthcare Common Procedure Coding System codes in any of the other Medicare files. These drugs were abarelix, bicalutamide, buserelin, cyproterone, degarelix, diethylstilbestrol, estramustine, flutamide, gonadorelin, goserelin, histrelin, leuprolide, medroxyprogesterone, megestrol, nafarelin, nilutamide, polyestradiol, or triptorelin. Ketoconazole was not included in this list of drugs because it is rarely used for androgen deprivation therapy in the United States. Evidence that the prostate cancer became resistant to surgical castration or androgen deprivation therapy was indicated by use of second-line systemic treatments (docetaxel, abiraterone acetate, sipuleucel-T, mitoxantrone, enzalutamide, or cabazitaxel) in Medicare data [5, 8, 9].

Eligible subjects were also required to be enrolled in both Medicare Parts A and B for at least 1 year before the cohort entry date and continuously between the date of initial diagnosis of prostate cancer and the cohort entry date.

Excluded were men who were enrolled in a health maintenance organization (HMO) during the year before cohort entry, had a diagnosis of any other cancer (except melanoma) on or before the cohort entry date, had any diagnostic code for metastases (other than bone or lymph node metastases) on or before the cohort entry date, had any claim for treatment with radium-223 on or before the cohort entry date, or had a claim for any second-line systemic therapy on or before the earliest date of surgical castration or androgen deprivation therapy (see Table 1). For the reason outlined in the Introduction, this study focused on patients who were not treated with radium-223. Radium-223 was originally approved by the FDA on May 15, 2013. It could have been prescribed in the US only during approximately the last six months of Medicare claims used in this study. Therefore, we planned to exclude any patient who received radium-223 on or before the date he otherwise would have been eligible for inclusion.

Cohort entry date was defined as the day on which the patient was identified as having CRPC (i.e., the date on which the patient first received a therapy representing a second-line systemic treatment for prostate cancer). Baseline comorbidities were identified based on at least one claim indicating another disease diagnosis on or before the cohort entry date. For each patient, follow-up continued until the earliest occurrence of death, discontinuation of coverage, claim for radium-223 treatment, or end of the study period. Follow-up for patients who disenrolled in either Medicare Part A or Part B or enrolled in an HMO was censored on that date for the survival analyses, and those patients did not contribute any additional follow-up time or events for incidence rate calculations.

SPM were ascertained using both SEER and Medicare data. In SEER data, the SPM was identified when there was any diagnosis of a primary cancer other than prostate after cohort entry. In Medicare data, an SPM was identified as an International Classification of Diseases, Ninth Revision, Clinical Modification code, for a primary malignancy other than nonmelanoma skin cancer or prostate cancer associated with one hospitalization, or with two hospital outpatient visits, or with two physician visits. In the inpatient file, the first such diagnosis occurring after the date of cohort entry was counted as the SPM event. In the outpatient or physician files, the second code for SPM (on a different date than the first) was counted as the SPM event. The strategy of using one inpatient or two outpatient or physician claims is consistent with methodology used by the Centers for Medicare and Medicaid Services (CMS) Chronic Condition Data Warehouse (CCW) [10] and with previous studies of SPM using SEER-Medicare data [2, 3].

### 2.4. Analysis

Descriptive analyses of the data were performed using summary statistics for continuous and categorical data. Tables with frequencies and percentages were generated for categorical data, but categories were combined or results were suppressed to avoid reporting any cell counts less than 11, as required by the SEER-Medicare Data Use Agreement.

The SPM incidence rate was estimated as the count of patients with an SPM divided by the person-years at risk among all patients (multiplied by 100 to express rates per 100 person-years). The Poisson distribution was used to estimate 95% confidence intervals (CIs). The proportion of patients with evidence of bone metastasis at cohort entry was calculated by dividing the number of patients with either bone metastasis (identified by diagnosis code) or bone-directed treatment (identified by a drug or treatment code) by the total number of patients in the study cohort. The Kaplan-Meier method was used to estimate the survival function for cohort members from the date of diagnosis of CRPC. Death was censored for patients who were still alive on the last day of the study period.

There was no ability to query to resolve missing values in the SEER-Medicare data, and no data imputations were performed. For indicator variables (e.g., presence or absence of a characteristic), if the patient was not recorded as having a given characteristic, the characteristic was assumed in the analysis not to be present in that patient. Counts of missing values were reported when summarizing categorical variables, and relative frequencies were based on all patients (including those with missing values).

Because there is limited published information on the reliability of Medicare claims data to identify second primary cancer outcomes, particularly in men with CRPC, we also assessed the effect of varying requirements for defining SPM ranging from less restrictive (a single claim in any Medicare file or a SEER diagnosis) to more restrictive (a SEER diagnosis only) criteria.

There was no internal comparison group for this study. To provide additional context, we compared our results to the expected incidence of all cancers (except prostate cancer) among men in the general population aged 65 or older (in the entire SEER database [11]) by estimating a standardized incidence ratio with 95% CI. This was done by applying the age-specific person-time (in 5-year age groups) from the present study to the SEER 1975-2013 age-specific annual incidence rates for all cancers [12] minus the rates for prostate cancer [13]. The expected incidence rate was then calculated by summing the expected number of cases for each age group and dividing by the total person-time in the present study.

## 3. Results

The SEER data contained 564,491 individuals diagnosed with prostate cancer from 2000 to 2011. Applying the inclusion and exclusion criteria resulted in a study cohort of 2,234 patients (see Table 1). No patient was excluded because of a claim for radium-223 treatment on or before his potential cohort entry date.

By design, all patients were aged 65 years or older, and the mean age at cohort entry was 76.6 years (Table 2). The study cohort was primarily white (83.6%), with the remainder black (9.8%), Asian (2.1%), or Hispanic (2.1%), or recorded as having another or unknown race (2.5%).

The numbers and percentages of patients and follow-up time stratified by year of cohort entry are listed in Table S-1. Year of cohort entry ranged from 2000 through 2013. The proportion of patients entered each year gradually increased over time.

At initial diagnosis, nearly 30% of prostate cancers were stage I/II (24.3%) or stage III (4.8%) (Table 3). Note that less than 0.5% were stage I, so categories were collapsed to prevent reporting cell counts less than 11. The remaining cases of known stage were stage IV (26.1%). Stage information was missing for 44.8% of the patients.

Most of the patients had a history of other serious medical conditions on or before the cohort entry date. The most common comorbidities (present in > 20% of patients) were chronic pulmonary disease (42.4%), diabetes without chronic complications (41.2%), peripheral vascular disease (37.2%), cerebrovascular disease (30.5%), congestive heart failure (28.5%), mild liver disease (22.9%), and renal disease (21.8%).

The diagnosis of metastases and use of bone-directed therapy were assessed in Medicare claims through all of the patients' available medical history and up to 30 days after the cohort entry date (to allow diagnostic and therapeutic claims to be counted that closely followed clinical determination of castration resistance). The great majority (80.4%) had recorded claims for bone metastases, and 59.4% had received bone-directed therapy; 84.5% had either bone metastases or bone-directed therapy. Thirteen percent had a history of lymph node metastases recorded in claims data. Nearly all patients (97.7%) in the cohort had undergone medical castration.

The average time from initial diagnosis of prostate cancer to development of CRPC was 42 months, with only 15% of the cohort developing CRPC within 1 year of the initial diagnosis. The majority (62%) had an interval of more than 2 years from initial prostate cancer diagnosis to development of CRPC.

Among the 2,234 men in the cohort, docetaxel was the most frequently identified (76%) second-line systemic therapy used to indicate CRPC, followed by abiraterone acetate (9.6%) and sipuleucel-T (8.5%) (Table 4).

Follow-up was censored at any time after cohort entry for 49 patients (2.2%) because of either HMO enrollment or discontinuation of Medicare Part A or B coverage. Among these, 37 (76%) had gaps in eligible coverage of longer than 3 months.

Treatments received after cohort entry included chemotherapy (94.9%), radiation therapy (32.5%), and radiopharmaceuticals (4.6%). Altogether, 172 cases of SPM were identified, yielding an incidence rate of 5.9 per 100 person-years (95% CI, 5.0-6.8) (Table 5). Using only SEER data alone, 20 cases of SPM were identified.

The most common second primary cancers were lung/bronchus (n = 29, 16.9%), urinary bladder (n = 22, 12.8%), colon/rectum (n = 21, 12.2%), nonprostate, nonbladder genitourinary tract (n = 18, 10.5%), and noncolorectal gastrointestinal (n = 17, 9.9%) (Table S-2).

Among the 172 patients with CRPC who developed an SPM, the mean (standard deviation) time between cohort entry and the diagnosis of the second cancer was 1 year (1.1).

Three-quarters of the patients (1,689 of 2,234) in the study died during follow-up. The median survival time after cohort entry was 1.2 years (95% CI, 1.1-1.3), and the survival probabilities at 1, 3, and 5 years were 56% (95% CI, 54-58%), 17% (95% CI, 15-18%), and 9% (95% CI, 7-11%), respectively.

The population-based incidence rate of all malignancies except prostate cancer in SEER for men in the general population aged 65 or older is 1.9 per 100 person-years (standardized incidence ratio = 3.1, 95% CI, 2.8-3.6).

## 4. Discussion

In the study cohort of patients with CRPC identified in SEER-Medicare data, the incidence rate of SPM was 5.9 per 100 person-years (95% CI, 5.0-6.8). Most men with CRPC (84.5%) either had a history of bone metastases or were prescribed a bone-targeting therapy. The rate of SPM we estimated is approximately 3 times higher than the population-based rate of all cancers (except prostate cancer) in SEER. Median survival time was relatively short, which is expected to limit the time at risk for development of SPM in men treated with new agents for CRPC.

One previous study in men with prostate cancer using SEER-Medicare data [3] evaluated only one SPM, colorectal cancer. Using only SEER outcome data, the incidence was 6.3 per 1,000 person-years (95% CI, 5.3-7.5) in men who had orchiectomies and 4.4 per 1,000 person-years (95% CI, 4.0-4.9) in those who were treated with gonadotropin-releasing hormone agonists. The investigators also conducted a sensitivity analysis additionally using Medicare documented cases and alluded to a discrepancy with their main analysis, but specific results were not reported. Also, since our study included only men with prostate cancer who had developed castration resistance, the results of that study should not be compared directly with ours.

Another study among cancer survivors of all ages in SEER reported an incidence rate of 81 per 1,000 for SPM [2]. The study included patients diagnosed with the 10 most common first cancers (prostate, breast, lung, colon, rectum, bladder, uterus, kidney, melanoma, and non-Hodgkin lymphoma) who were followed up for a minimum of 3 years. These results also cannot be directly compared with ours because the Donin [2] study included younger patients with a variety of first cancers and used only SEER data to assess SPM, and the survival of patients (mean follow-up 7 years) was much longer than in our study (in which only 9% of patients were alive after 5 years). We are not aware of any previous study that has estimated the incidence of SPM among men with CRPC specifically.

The SEER-Medicare database population is considered representative of the general US population and is the largest available source of detailed population-based medical information on men aged 65 years or older with prostate cancer. Therefore, this study should provide more precise estimates of SPM risks in men with CRPC than any other available US data resource. We did not need to use SEER data on stage of cancer at initial prostate cancer diagnosis to define the study population, which is an additional strength of the design since such data were missing for nearly 45% of the included patients. Thus, the results apply broadly to the population of patients who received a second-line treatment after medical or surgical castration. However, because outcomes might vary among subgroups of patients—for example, those with metastatic disease at the time of their initial prostate cancer diagnosis—the results should not be assumed to reflect similar outcomes among all possible subgroups of the study population.

As stated by NCI, no algorithm accurately and completely identifies patients with metastases in SEER-Medicare claims [15, 16]. We can assume that these codes have suboptimal sensitivity, so the true number of cases with metastases likely exceeds the number we have reported. NCI cautions that these codes should be used selectively. Therefore, these codes were not used in our study for the primary analysis but instead to provide further context for our results.

We used second-line treatment (after surgical castration or medical androgen deprivation therapy) to define CRPC because the biochemical and diagnostic radiologic data necessary to diagnose castration resistance in routine clinical practice are not available in claims data (see Section 20.1 in Mottet et al. [17]). There are likely additional patients who are diagnosed with CRPC (i.e., they would have met biochemical and clinical criteria for CRPC) in the SEER-Medicare database who were not eligible for this study because they did not receive a second-line systemic treatment. Although this may be seen as a limitation, it could also be considered a strength of the study design if the results are intended to provide context for estimates of SPM incidence rates among patients with CRPC who are given a second-line systemic treatment.

A potential limitation of this study was that several of the drugs used to identify medical castration are oral therapies that might have been identifiable only among patients who had Medicare Part D coverage. This would have resulted in some potentially eligible patients not being included in the study cohort. However, almost 90% of patients in our study were identified using both Part D and non–Part D data, and only a small proportion was identified only in Part D data (results not shown). Another potential limitation is that to simplify the analysis, follow-up time was censored after patients enrolled in an HMO or discontinued Medicare Part A or B coverage. If interruptions in coverage were associated with poorer health outcomes, this could have resulted in underestimation of the incidence of SPM or overestimation of survival. However, since the number of patients affected by changes in insurance coverage during follow-up was so small (2.2%), the alternative analytic method of allowing a portion of these patients' follow-up time (after reenrollment in eligible coverage) to be considered in the analysis would likely have had a negligible effect on the results.

Results from studies using US federal insurance data to supplement SEER data to identify cancer in older Americans depend on criteria used to define cases [18]. Most SPM in our study were identified only in Medicare data. Given these findings, investigators should be aware that SEER-Medicare data may yield varying estimates of SPM depending on case identification criteria. Lower incidence rates are likely to be estimated using SEER-registered diagnoses of SPM than using both SEER and Medicare data files. Imposing restrictions on the temporal relation between the two outpatient or physician diagnoses of a second cancer (other than that they not occur on the same date [10]) would have resulted in fewer cases and a lower incidence estimate. Sensitivity analyses can be useful to understand the extent of differences in case identification with varying criteria [19]. In addition, the relatively high frequency of bladder and other genitourinary cancers found in Medicare data suggests the possibility that local spread of advanced prostate cancer may in some instances have been recorded as SPM. In other words, some SPM found in the Medicare data may be false positives (not true SPM).

## 5. Conclusions

The observed incidence rate in this study is approximately threefold higher than expected from population-based incidence rates in SEER among similarly aged men without prostate cancer. This highlights the importance of estimating baseline incidence rates of specific conditions, such as SPM, to provide context for findings from postmarketing safety studies in populations for whom new therapies are being introduced.

## Figures and Tables

**Table 1:** Cohort selection.

Reason for exclusion	No. of patients (%)	Remaining sample
Initial sample of prostate cancer cases from SEER-Medicare data	564,491 (100)	564,491
No record of surgical or biologic castration	383,713 (67.98)	180,778
No record of second-line systemic therapy^†^ after castration date	168,388 (29.83)	12,390
Castration was on or before prostate cancer diagnosis date	376 (0.07)	12,014
Diagnosis of any cancer other than prostate cancer or nonmelanoma skin cancer on or before potential cohort entry date	5,543 (0.98)	6,471
Diagnostic code for exclusionary metastases (197X or 198X with exception of 198.2-skin or 198.5-bone) on or before potential cohort entry date	1,767 (0.31)	4,704
Not aged at least 65 years on potential cohort entry date	246 (0.04)	4,458
Not continuously enrolled in both Parts A and B Medicare coverage between the earlier of (1) 12 months before cohort entry or (2) the month of prostate cancer diagnosis and cohort entry date	1,293 (0.23)	3,165
Enrolled in HMO either (1) in year before potential cohort entry date, or (2) at some time between diagnosis date of initial prostate cancer identified in SEER and potential cohort entry date	931 (0.16)	2,234

SEER, Surveillance, Epidemiology, and End Results program of the US National Cancer Institute; HMO, health maintenance organization.

^†^ Abiraterone, cabazitaxel, docetaxel, enzalutamide, mitoxantrone, or sipuleucel-T.

**Table 2:** Demographic characteristics of study cohort (N = 2,234).

**Variable**	**No. of patients (**%**)**
**Age at cohort entry, years**	
Mean (SD)	76.6 (6.2)
Age group	
65-69	297 (13.3)
70-74	625 (28.0)
75-79	595 (26.6)
80-84	451 (20.2)
85+	266 (11.9)
**Race**	
White	1,867 (83.6)
Black	218 (9.8)
Asian	46 (2.1)
Hispanic	48 (2.1)
Other or unknown^†^	55 (2.5)

SD, standard deviation.

^†^ Categories were combined to avoid reporting a count of < 11.

**Table 3:** Clinical characteristics of study cohort (N = 2,234).

Variable	No. of patients^†^ (%)
**Characteristics at initial prostate cancer diagnosis**	
Stage (derived group)^‡^	
Stage I or II^§^	543 (24.3)
Stage III	107 (4.8)
Stage IV	583 (26.1)
Unknown	1,001 (44.8)
**Characteristics on or before cohort entry date**	
Comorbidities_ _^¶^	
Chronic pulmonary disease	947 (42.4)
Diabetes without chronic complications	920 (41.2)
Peripheral vascular disease	830 (37.2)
Cerebrovascular disease	681 (30.5)
Congestive heart failure	636 (28.5)
Mild liver disease	512 (22.9)
Renal disease	487 (21.8)
Myocardial infarction	359 (16.1)
Diabetes with chronic complications	273 (12.2)
Rheumatic disease	183 (8.2)
Peptic ulcer disease	171 (7.7)
Paraplegia and hemiplegia	87 (3.9)
Dementia	83 (3.7)
Moderate or severe liver disease	18 (0.8)
AIDS/HIV	< 11
Metastases^††^	
Lymph node	296 (13.2)
Bone	1,797 (80.4)
Bone-directed therapy^††^	1,326 (59.4)
Either bone metastases or bone-directed therapy	1,887 (84.5)
Castration method	
Surgical	52 (2.3)
Medical	2,106 (94.3)
Surgical and medical	76 (3.4)
Time from initial diagnosis to development of CRPC	
Mean (SD), months	42.1 (32.6)
Distribution	
< 6 months	89 (4.0)
6 months to 1 year	251 (11.2)
> 1 to 1.5 years	279 (12.5)
> 1.5 to 2 years	223 (10.0)
> 2 years	1,392 (62.3)

AIDS, acquired immune deficiency syndrome; HIV, human immunodeficiency virus; CRPC, castration-resistant prostate cancer; SD, standard deviation.

^†^ Unless stated otherwise.

^‡^ Stage according to the *AJCC Staging Manual, Sixth Edition* [14].

^§^ Categories were combined to avoid reporting a count of < 11.

^¶^ Individual patients can have multiple comorbidities; thus, the sum of all comorbidities adds up to more than 100%.

^††^ Recorded anytime between initial date of prostate cancer diagnosis and 30 days after the cohort entry date.

**Table 4:** Second-line therapies indicating castration-resistant prostate cancer (N = 2,234).

Therapy	n	%
Docetaxel	1,697	76.0
Abiraterone acetate	215	9.6
Sipuleucel-T	191	8.5
Mitoxantrone	86	3.8
Enzalutamide	30	1.3
Cabazitaxel	15	0.7

**Table 5:** Incidence rates of second primary cancer, per 100 person-years.

Case identification	Patients	Person-years	Cases	Rate (95% CI)
SEER and Medicare	2,234	2,922	172	5.9 (5.0-6.8)
Age at cohort entry, years				
65-69	297	551	30	5.4 (3.7-7.8)
70-74	625	920	63	6.8 (5.3-8.8)
75-79	595	747	37	5.0 (3.5-6.8)
> 80^†^	717	704	42	6.0 (4.3-8.1)
SEER only	1,664	2,055	20	0.97 (0.59-1.5)

CI, confidence interval; SEER, Surveillance, Epidemiology, and End Results program of the United States National Cancer Institute.

^†^ Categories were combined to avoid reporting a count of less than 11.

## Data Availability

This study was conducted using data from NCI's SEER program of the United States and guided by a data use agreement between NCI and RTI Health Solutions. It was reviewed by the RTI IRB and received an exemption on February 23, 2016.

## Supplementary Materials

Supplementary Materials Table S-1. Study patients and follow-up years, by cohort entry year (N = 2,234). Table S-2. Types of second primary malignancies found using Surveillance, Epidemiology, and End Results (SEER)-Medicare data.
