# Corrigendum to “Incidence of Second Primary Malignancies in Patients with Castration-Resistant Prostate Cancer: An Observational Retrospective Cohort Study in the United States”

**DOI:** 10.1155/2019/3425982

**Published:** 2019-07-04

**Authors:** Catherine W. Saltus, Zdravko P. Vassilev, Jihong Zong, Brian Calingaert, Elizabeth B. Andrews, Montse Soriano-Gabarró, James A. Kaye

**Affiliations:** ^1^RTI Health Solutions, Waltham, Massachusetts, USA; ^2^Bayer US, Whippany, New Jersey, USA; ^3^RTI Health Solutions, Research Triangle Park, North Carolina, USA; ^4^Bayer AG, Berlin, Germany

In the article titled “Incidence of Second Primary Malignancies in Patients with Castration-Resistant Prostate Cancer: An Observational Retrospective Cohort Study in the United States” [1], there was an error in the “2.3. Study Design and Subjects” section, where “(except melanoma)” should be corrected to “(except nonmelanoma skin cancer).”
