# Supplementary material for: Incidence of Second Primary Malignancies in Patients with Castration-Resistant Prostate Cancer: An Observational Retrospective Cohort Study in the United States
Source: Prostate Cancer. 2019 Feb 11;2019:4387415. doi: 10.1155/2019/4387415 (PMC6388338; doi:10.1155/2019/4387415)
Supplement: Supplementary Materials — Table S-1. Study patients and follow-up years, by cohort entry year (N = 2,234). Table S-2. Types of second primary malignancies found using Surveillance, Epidemiology, and End Results (SEER)-Medicare data. [file 4387415.f1.pdf]

SUPPLEMENTARY MATERIAL

Table S-1 Study Patients and Follow-up Years, by Cohort Entry Year (N = 2,234)

Cohort Entry Year	Patients n (%)	Follow-up, Years n (%)	Follow-up, Years per Patient	
			Mean (SD)	Five-Number Summary (Min-Q1-Median-Q3-Max)
2000/2001†	51 (2.3)	89 (3.0)	1.74 (3.09)	0.03 - 0.26 - 0.72 - 1.58 - 13.45
2002	67 (3.0)	120 (4.1)	1.78 (2.94)	0.00 - 0.35 - 0.72 - 1.71 - 11.98
2003	84 (3.8)	114 (3.9)	1.36 (1.81)	0.01 - 0.41 - 0.90 - 1.58 - 10.90
2004	118 (5.3)	198 (6.8)	1.68 (1.99)	0.07 - 0.46 - 1.01 - 1.87 - 9.35
2005	179 (8.0)	319 (10.9)	1.78 (2.19)	0.03 - 0.53 - 0.97 - 2.02 - 8.91
2006	173 (7.7)	257 (8.8)	1.49 (1.53)	0.00 - 0.46 - 1.06 - 1.72 - 7.99
2007	188 (8.4)	309 (10.6)	1.65 (1.62)	0.03 - 0.52 - 1.00 - 2.19 - 6.80
2008	164 (7.3)	232 (7.9)	1.41 (1.35)	0.00 - 0.52 - 1.05 - 1.84 - 5.99
2009	192 (8.6)	281 (9.6)	1.47 (1.24)	0.02 - 0.55 - 0.95 - 2.19 - 4.82
2010	192 (8.6)	239 (8.2)	1.24 (0.98)	0.01 - 0.50 - 1.00 - 1.56 - 3.94
2011	256 (11.5)	335 (11.5)	1.31 (0.86)	0.02 - 0.53 - 1.21 - 2.07 - 2.98
2012	292 (13.1)	295 (10.1)	1.01 (0.53)	0.02 - 0.53 - 1.12 - 1.43 - 1.99
2013	278 (12.4)	134 (4.6)	0.48 (0.29)	0.00 - 0.22 - 0.46 - 0.74 - 0.99

Abbreviations: max, maximum; min, minimum; SD, standard deviation; Q1, first quartile; Q3, third quartile.

†Data for the years 2000 and 2001 were combined to prevent the reporting of cell counts less than 11, which is prohibited by the Surveillance, Epidemiology, and End Results (SEER)-Medicare data use agreement.

Table S-2 Types of Second Primary Malignancies Found Using Surveillance, Epidemiology, and End Results (SEER)-Medicare Data

Cancer Type	n	%
Lung/bronchus	29	16.9
Urinary bladder	22	12.8
Colon/rectum	21	12.2
Nonprostate, nonbladder genitourinary tract†	18	10.5
Noncolorectal gastrointestinal‡	17	9.9
Non-Hodgkin lymphoma and myeloma	15	8.7
Brain	14	8.1
Miscellaneous or unspecified	13	7.6
Meninges, head, neck, and endocrine	12	7.0
Melanoma, breast, and nipple	11	6.4
Total	172	100

†Nonprostate, nonbladder genitourinary tract = cancers of the kidney, ureters, urethra, and testis.

‡Noncolorectal gastrointestinal = cancers of the esophagus, stomach, small intestine, liver, biliary tract, and pancreas.
